# Longitudinal Trends in Sport Participation and Retention of Women and Girls

**DOI:** 10.3389/fspor.2020.00039

**Published:** 2020-04-16

**Authors:** Rochelle Eime, Jack Harvey, Melanie Charity, Hans Westerbeek

**Affiliations:** ^1^School of Health and Life Sciences, Federation University, Ballarat, VIC, Australia; ^2^Institute for Health and Sport, Victoria University, Melbourne, VIC, Australia

**Keywords:** women and girls, sport, sport club, drop-out, adolescents, retention

## Abstract

Measurement and analysis of sport participation data is vital to understand trends, and therefore to make informed decisions relating to sport policy and strategies to get more people active through sport. This study identified patterns of club sport participation, retention and drop-out of women and girls over a 7 year period in a popular team sport in Australia. The study included registered women and girls of all ages (4–96 years at baseline) in an almost exclusively female sport, with a particular focus on the ages 4–14 years where most participation occurs. All commencing participants in the base year (2010) were tracked over the 7 year period. Participants were classified in two ways: the total number of years they played and their overall pattern of participation. Differences between age groups were analyzed using Kruskall Wallis and Mann Whitney tests. Registration records of 29,225 participants were analyzed in the study. Overall, there were considerable differences in the years participating in the sport. Almost one third (30%) of commencing 4–9 year olds played continuously for the 7 years. This proportion diminished through ages at commencement from 10 to 19, reaching a low point of 4% for ages 15–19, then rebounded slightly, reaching 7% for ages 30+. The proportion who dropped out during the 7 year period and did not return varied with age in the converse manner, as did the proportion of single-year players. The optimal age of entry to sport for retention in participation was 6–9 years. Consideration needs to be given to the age appropriateness of sports programs for very young participants. Strategies specifically relating to retention of girls and young women during adolescence should be developed.

## Introduction

Participation in leisure-time physical activity (LTPA) is important from a public health perspective. An important component of LTPA is sport, a term which in the Australian context is usually reserved for competitive activities undertaken in community club settings (Eime et al., 2013a). Participation in organized sport makes an important contribution to overall LTPA levels across the lifespan (Olds et al., 2009; Makela et al., 2017). Furthermore, there is evidence that participation in club-based and particularly team-based sport can be associated with better psychosocial health than individual activities, due to the social nature of participation (Eime et al., 2013b).

However, while there is an abundance of research on LTPA, this research has tended to focus on overall levels of physical activity, and not on sport specifically (Eime et al., 2001; Coll Cd et al., 2014). Understanding sport participation, including retention and drop out at all ages across the lifespan, is important to inform policies and strategies to promote lifelong participation which are priorities of both sport management and public health domains (Australian Sports Commission, 2015). In the specific context of the Australian sporting landscape it is interesting and important to observe that Sport Australia—the peak government governing body for all sport—in its 2018 launch of the national sport plan “Sport 2030,” included the reduction of physical inactivity as one of four strategic objectives (Department of Health, 2018). Therefore, the facilitation of physical activity for children and their continued involvement is within the remit of Sport Australia and sports organizations. In line with this, the development of fundamental movement skills for children is essential (Department of Health, 2018).

We know that participation in sport during adolescence is associated with higher physical activity levels in adulthood (Bélanger et al., 2015). However, studies examining sport participation have suggested that sport drop-out (attrition) is relatively high, particularly during adolescence (Olds et al., 2009; Brooke et al., 2014; Eime et al., 2016a). There is evidence that adolescents play fewer sports than in childhood, and many drop out of sport completely (Eime et al., 2019b). Further, there are reports that adolescents (Pate et al., 2007; Eime et al., 2013a; Coll Cd et al., 2014) and adults (Klostermann and Nagel, 2014) are tending to shift their participation away from organized and competitive modes to non-organized and non-competitive modes and settings, and from team-based to individually based activities. A retrospective study conducted in the United States reported that 94% of Grade 10 students had withdrawn from at least one sport since Grade 1 and 30% had permanently withdrawn from sport participation (Butcher et al., 2002). Children tend to sample sports when they are young, and later focus their participation in a single sport as they age (Coté and Hay, 2002). This may explain high withdrawal rates from sport reported in some studies (Butcher et al., 2002). It has also been suggested that opportunities for organized sport participation decline as young people age because competitive success tends to be prioritized and therefore there are fewer opportunities for those less competent to play sport (Kirk, 2005). For example, many schools have only one team in each sport at each grade level and therefore competence plays a crucial part in determining whether a student will make the team (Butcher et al., 2002). Others suggest that sport participation drop-out is offset by a compensating tendency to participate in other leisure-time physical activities (Downward et al., 2014). It is also likely that the prioritization of elite performance over mass participation in state and national sport policies and associated funding (Nicholson et al., 2010) contributes to the lack of sport opportunities for adolescents and adults. There is also evidence that the drop-out for children (over a 4 year period) is not consistent across leisure-time activities (Brooke et al., 2014).

The reported drop in sport participation by children and adolescents appears to occur at around the same age that opportunities to enter elite pathways occur. Various theories and models assist in understanding the different sport participant pathways, both across the lifespan generally and for particular transitions such as into elite participation (Coté and Hay, 2002; Gulbin et al., 2013; Williams, 2013). For example, the Sport Commitment Model (Williams, 2013) identifies six determinants of sport commitment including sport enjoyment, involvement opportunities/benefits, personal investments, social support, involvement or attractive alternatives, and social constraints. Involvement or attractive alternatives relates to the attractiveness or appeal of other activities (Williams, 2013).

The Developmental Model of Sport Participation developed by Coté and Hay (2002) provides a framework to account for the different pathways of involvement in sport including recreational participation and elite performance. The sampling phase occurs between the ages of 6 and 12 years when children participate in a variety of sports and are involved in a high amount of deliberate play activities (Coté et al., 2009). The sampling phase develops fundamental motor skills and sport-specific skills while keeping children interested and motivated in sport (Coté, 2005). The recreational years (ages 13+ years) are an extension of the sampling years where children continue to participate in sport for enjoyment; whereas the specializing (13–15 years) and investment years (16+ years) apply to youth interested in more performance-oriented pathways, where the focus moves from deliberate play to deliberate practice activities (Coté and Hay, 2002). Sport sampling in children should be promoted as it is linked to higher physical activity levels during adolescence (Gallant et al., 2017).

In specific relation to the participation and drop-out of women and girls, there is some evidence that playing sport in a context or environment where males also participate (and witness) women and girls playing sport may lead to heightened self-awareness and anxiety, in turn leading to dropping out of active sport participation (Evans, 2006; Craike et al., 2009).

In summary, in relation to sport we know that: (a) many children sample many different sports before choosing one or two in which to specialize (Gallant et al., 2017; Eime et al., 2019b); (b) participation decreases with age (Coll Cd et al., 2014; Eime et al., 2016b); (c) boys are more likely to participate than girls and girls may drop out early because of not wanting to play sport in presence of boys/men (Casey et al., 2019); (d) many drop out of participation during adolescence, when the elite pathway begins for those most talented (Coté et al., 2009); and (e) more people are transitioning from organized sport to un-organized forms of participation (Eime et al., 2010; Vella et al., 2014). Although there are other reasons for drop-out, a spike in leaving organized sport (out of sport altogether or into unorganized sport or physical activity) can be observed just when the elite pathway begins for the most talented. Nevertheless, we know little about the longitudinal nature of participation, retention and drop out across age groups.

There is a general absence of robust sport participation data to inform national sport policy and strategic development (Eime et al., 2001; Balaska et al., 2014). Most nationally and internationally representative studies of sport participation are cross-sectional (Eime et al., 2001; Downward and Rasciute, 2010; Downward et al., 2014). Furthermore many sport drop-out studies are retrospective (Butcher et al., 2002) or limited to specific age-based cohorts (Telama et al., 2006; Richards et al., 2007). Longitudinal studies, with detailed analysis of patterns of sport participation, retention and attrition across the lifespan, are required to better inform sport management policy and practice in contexts ranging from community “grass-roots” participation to elite levels of competition.

Similarly, given the lack of national sport participation data in Australia, it has been recently advocated that utilizing sport-specific data collected by sports organizations may provide a more detailed evidence-base to facilitate a better understanding of the extent of sport participation, retention and withdrawal, and thereby inform strategies in program and policy development in sport (Eime et al., 2016a). Furthermore, it has been suggested that sport participation analysis requires “a more detailed analysis of age, cohort and period effects based on complete cohort-sequence plans” (Klostermann and Nagel, 2014, p. 631.)

Therefore, the purpose of this study was to identify patterns of club sport participation, retention and drop-out over a 7 year period among women and girls in a popular team sport in Australia. This study included participants of all ages across the lifespan, and also included a particular fine-grained focus on the ages 4–14 years where most participation occurs.

## Methods

This study drew on the registration records of women and girls participating in an almost exclusively female club-based team sport in the Australian state of Victoria between 2009 and 2016. The sport, which as a condition of using its data cannot be identified, was chosen for study because of its comprehensive repository of consistent and reliable registration data over an 8 year period. Other factors influencing the choice were fact that the rate of participation in sport by women and girls is half that of boys and men (Eime et al., 2019a), and the avoidance of mixed gender contexts where boys and men (negatively) influence the sense of skill, competence or body image of women and girls (Evans, 2006; Casey et al., 2009; Craike et al., 2009). This sport was ranked within the top 10 organized physical activities and regular club-based physical activities in Australia (Australian Bureau of Statistics, 2013). The study was approved by the University Human Research Ethics Committee.

A participant was defined as a participant registered with the sport's state governing body. In the context of community sport in Australia, the vast majority of those registered are active participants (players), and so registration provides an excellent proxy for active participation (Eime et al., 2016a,b). Measures of duration of participation are most meaningful if participation is tracked from the year of commencement. This was achieved by using the second year of available data (2010) as the base year, and using the first year of available data (2009) to exclude from the study any participant registered in the base year (2010) who had also been registered in the previous year (2009). In this way, only those for whom the base year of the study (2010) was their first year of participation (including possible re-commencement after a break in participation) were included in the study. These participants were then tracked from the base year (2010) through a 7 year period to 2016.

While the data were longitudinal in nature, with a panel or cohort consisting of all commencing participants in the base year, the aim of this study was different from the usual panel study in which the investigation is focused on the changes over time in some characteristic measured at each time point. The characteristic of interest in this study was dichotomous—participation or no participation. But rather than investigating changes from each year to the next, we analyzed two indicators—one quantitative and one categorical—derived from the pattern of participation of each individual over the 7-year period. The indicators were (a) the total number of years played during the 7 year period; and (b) the overall pattern of participation throughout the 7 years. Each of these indicators was compared for age groups based on age in the base year. There are thus two aspects: the 7 year longitudinal indicators, and the comparison of these indicators for different cohorts based on age at baseline (2010).

The first indicator (total number of years played) is self-explanatory. Kruskall Wallis and Mann Whitney tests were used to identify significant differences in the total years played according to the initial age of the player.

The second indicator (pattern of participation) bears on the concepts of retention and drop-out, both from year to year and over longer periods. In this study, the only point of commonality is that all players in the study participated in the base year (2010). There are 64 (=2^6^) possible patterns of participation over the succeeding 6 year period. Players were classified into one of three categories based on the overall pattern of their involvement over this period, as follows:

Continuous participation for the 7 year period (1of 64 patterns);
Drop-out with no return during the 7 year period (6 of 64 patterns—drop-out after any of years 1–6).
Intermittent participation, with gaps, i.e., drop-outs and returns, during the 7 year period (57 of 64 patterns);

Participation profiles for both indicators were produced for all players in 5 multi-year age cohorts and for single-year age cohorts for the 4–14 age range.

## Results

**Figure 1:**
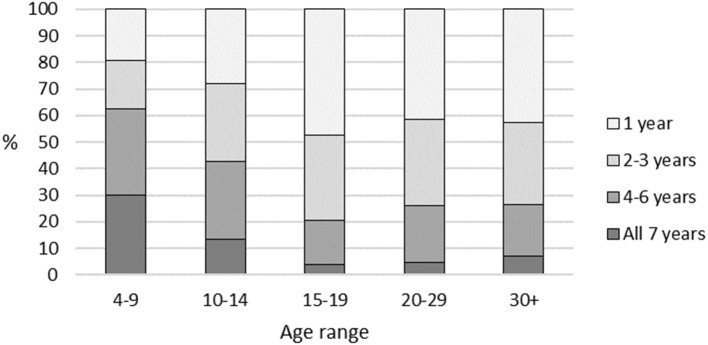
Total years of participation: profiles by age group.

Registration records of 29,225 participants were analyzed in the study. Table 1 shows profiles of the number of years playing the sport, for all participants, and for each age group. Table 1 and Figure 1 display percentages of the number of participants in each age group, to provide comparative profiles of participation patterns. Participation for the whole 7 year period was highest amongst the youngest participants who commenced aged 4–9 years, with a steady decline in all-years participation through to commencement ages 15–19. From commencement at age 4–9 through to 15–19 the proportion participating for only 1 year increased from 20 to 48%, and remained over 40% for all older age groups.

**Table 1:** Total years of participation: profiles and summary statistics by age group.

	**Age group (years)^a^**					
	**4–9**	**10–14**	**15–19**	**20–29**	**30+**	**All ages**
**Total number of years played**	**%**	**%**	**%**	**%**	**%**	**%**
1	19	28	47	41	43	32
2	10	17	20	21	19	16
3	9	12	12	12	12	11
4	9	11	7	8	8	9
5	10	9	5	7	6	8
6	13	8	5	6	5	9
7	30	13	4	5	7	15
N	9,478	7,090	3,064	5,151	4,442	29,225
Median	5	3	2	2	2	3
Mean	4.4	3.4	2.3	2.6	2.6	3.3

a *Age at baseline (2010)*.

Table 1 also shows median and mean number of years played. A Kruskal Wallis test established that there were significant differences between the number of years played by each age group (*p* < 0.001). Mann Whitney *post-hoc* pairwise comparisons between the age groups established that, with the exception of the pair 20–29 and 30+, all other pairwise differences between age cohorts were significant (*p* < 0.001). These tests have very high statistical power and sensitivity because of the very large sample sizes.

**Figure 2:**
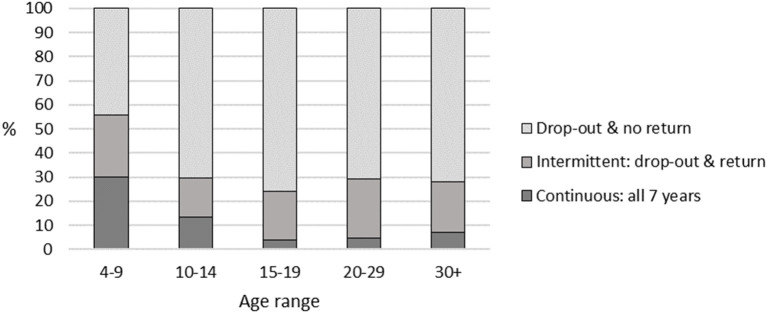
Patterns of participation: profiles by age group.

Table 2 and Figure 2 provide a breakdown by age groups of the three categories of participation pattern across the 7 years. The rate of continuous participation was highest amongst 4–9 year old commencers (30%), and steadily declined until the 15–19 year age group, which had the lowest rates of continuous participation (4%). The rate of complete drop-out, i.e., drop-out with no return, was lowest amongst 4–9 year old commencers (44%), and over 70% in all other age groups, the highest rate being 76% for the 15–19 year age group. The rate of intermittent participation throughout the 7 years was relatively stable across all age groups, with rates between 16% (ages 10–14 years) and 26% (ages 4–9 years).

**Table 2:** Patterns of participation: profiles by age group.

	**Age group (years)^a^**					
	**4–9**	**10–14**	**15–19**	**20–29**	**30+**	**All ages**
**Pattern of participation**	**%**	**%**	**%**	**%**	**%**	**%**
Continuous: all 7 years	30	13	4	5	7	15
Intermittent: drop-out and return	26	16	20	25	21	22
Drop-out and no return	44	71	76	71	72	63
N	9,478	7,090	3,064	5,151	4,442	29,225

a *Age at baseline (2010)*.

**Figure 3:**
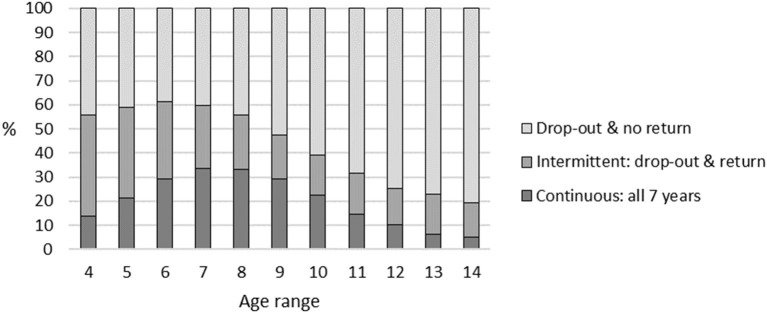
Patterns of participation: profiles by age in single years (4–14).

Table 3 and Figure 3 provide further breakdowns of individual ages from 4 to 14 years. This demonstrates that initiating participation in this sport from age 6–9 resulted in the highest rates of continuous participation. The lowest rates of drop-out with no return occurred for commencers aged 5–7.

**Table 3:** Patterns of participation: profiles by age in single years (4–14).

	**Age (years)^a^**											
	**4**	**5**	**6**	**7**	**8**	**9**	**10**	**11**	**12**	**13**	**14**	**All ages**
**Pattern of participation**	**%**	**%**	**%**	**%**	**%**	**%**	**%**	**%**	**%**	**%**	**%**	**%**
Continuous: all 7 years	14	21	29	34	33	29	22	14	10	6	5	23
Intermittent: drop-out and return	42	38	32	26	23	18	17	17	15	16	14	22
Drop-out and no return	44	41	39	40	44	53	61	68	75	77	81	55
N	219	822	1,408	2,177	2,590	2,262	1,954	1,739	1,364	1,077	956	16,568

a *Age at baseline (2010)*.

## Discussion

There is an absence of large-scope national sport participation data to inform sport policy and practice, both in and outside Australia (Eime et al., 2001; Balaska et al., 2014) and therefore deep understanding of the trends in organized, club-based sport participation has been lacking (Eime et al., 2001). Many other studies are not specific to sport and incorporate all out of school activities such as household chores or walking the dog in addition to traditional sports such as netball and hockey (Brooke et al., 2014). The present study captured a large population of annual registration records longitudinally (over 7 years) to explore sport participation retention and withdrawal trends among women and girls across all age groups and in more detail for girls aged 4–14 years, who represent the highest proportion of participants. This information contributes to the knowledge of sport participation trends across the lifespan, extending existing research that was previously limited to those aged 15 years or older (Eime et al., 2001), and will help to inform strategic management of sport programs and policy developments with a view to optimizing the uptake of sport and maximizing continued sport participation.

Across the whole age range, 15% of players played continuously across the 7 years. Continuing participation peaked amongst the lowest age group 4–9 years, before decreasing to the lowest retention amongst 15–19 year olds. This is consistent with the results of previous research; whilst participation in sport is popular among children, there is considerable withdrawal especially during adolescence. Some of the withdrawal can be attributable to a trend away from participation in club- and team-based competitive sports (Vella et al., 2014). Adolescents, and particularly girls, shift their participation to more individual unstructured types of physical activity like walking and jogging as they age from 16 to 20 years (Eime et al., 2013a; Downward et al., 2014). Borgers et al. (2015) states that there is an increasingly diverse range of participation activity types and domains, and therefore the sport participation market requires diversification too, and should not be considered to be a single market.

Whilst other studies have investigated participation and drop-out in sport, few have also investigated discontinuous participation, that is, people who leave the sport but return. The highest rate of drop-out and return during the 7 year period was for those aged 4–9 years and 20–29 years at baseline. For the younger participants this would be likely due to sampling, whereby participants try a range of sports before specializing (Eime et al., 2019b). However, for the young adults it could be due to a range of factors including life transitions such as childbearing, and/or injury.

The decline in sport participation during adolescence aligns with the Developmental Model of Sport Participation, where participants move from sampling various sports into specialization (Coté and Hay, 2002). Furthermore, there may be a trend for people to transition into a non-competitive recreational phase instead of specializing in a competitive form of the sport (Coté and Hay, 2002). It may be that sport as it currently stands does not offer enough non-competitive formats of play after the modified sports for the very young, particularly for those with lower levels of competency (Eime et al., 2013a). The Developmental Model of Sport Participation identifies broad age ranges according to each stage of development (Coté and Hay, 2002). However other participation development pathway models, such as the Foundations, Talent, Elite and Mastery (FTEM) do not specify fixed age boundaries (Gulbin et al., 2013). The FTEM, like the Developmental Model of Sport Participation, acknowledges that participants may drop out of sport at any stage and/or age, or may remain in the sport-specific commitment and/or competition stage for a lifetime, either due to choice or ability (Gulbin et al., 2013). However, the results of the current study show a substantial decline in participation of young women in a particular predominantly female sport during late adolescence, which is not only a time of increase in competing priorities, including study, employment and social and romantic relationships (Eime et al., 2013a), but is also when the elite pathway is developing in many sports. Therefore, some of the drop-out in sport may be related to Australian national sport policies, where funding for sport was heavily skewed toward elite athletic performance rather than grass-roots participation (Australian Sports Commission, 2014). However, as noted earlier, there seems to be a policy transition toward (1) acknowledging the importance of a better resourced focus on grassroots participation in sport and (2) an extension of remit into facilitating participation in physical activity, and as such lowering the threshold to become involved in sport.

Whilst the highest retention rates were for the group aged 4–9 years, when further broken down into single years of age, the optimal starting age for higher retention may be between the ages of 6–9 years. It is evident that children are participating in organized sport earlier than previously, such as the reported age of “around seven or younger” by Coté and Hay (2002). This may be related to national sports policy which has a strong focus on increasing numbers of participants from year to year. Sporting organizations might have taken the view that recruiting young beginners is a simpler and/or more effective means of increasing numbers than promoting retention of existing participants by identifying and addressing the modifiable determinants of drop-out. It may also be that corporate sponsorship of introductory junior programs has a role in encouraging this approach. However, the high rates of attrition among older children and adolescents indicates that while such a strategy might be effective for increasing numbers in the short term, it does not build on the investment in the early years or produce the potentially life-long benefits of continued participation. It would also further strengthen the argument that exposure to non-competitive playful physical activity during the early childhood years would better prepare youngsters for structured sport participation later in childhood. Perhaps preschool aged children may not be developmentally ready for organized sport (Committee on Sports Medicine and Fitness and Committee on School Health, 2001). There is evidence that when the demands and expectations of the sport exceed the maturation or readiness of the child, then the potential benefits are offset, and that young children should still participate in free play and child-organized activities (Committee on Sports Medicine and Fitness and Committee on School Health, 2001).

The authors have previously recommended that sport policy have a specific focus on retention of participants and not simply on total increase in numbers each year (Eime et al., 2016a). Furthermore, sports organizations should strategically focus on recruitment strategies into sport for those aged 6–9 years and into fundamental movement skills programs for younger children (Eime et al., 2018). For those that are older (10+ years) when they enter the sport the lower retention rates may be due to a lack of sport-specific competency compared to other peers that may have previous experience. As noted, it does not seem that playing a female-only sport alleviates lack of perceived competency matter in regard to deciding to keep playing or dropping out of sport.

There is increasing attention on children and adolescent participation, but very few studies have examined sport participation patterns in adults (Lim et al., 2011). The low retention throughout late adolescence and early adulthood may be related to the life courses and transitional dynamics such as changes from youth to adulthood, from single to married, to becoming a parent and so on (Lim et al., 2011). For example, parents reported a need to provide opportunities for their children above the need for their own participation (Lim et al., 2011), or that time spent caring for their children constrains their own participation in sport (Ruseski et al., 2011). This knowledge, together with the frequently assumed dominant carer role by women and girls, may explain the much higher rates of discontinuous participation found in this study for women aged 15–60 years.

Most studies of sport participation published to date are based on relatively small samples who self-report their participation in sports. This study aligns conceptually to a degree with such studies, in that it is based on registered participants in a community sport, the overwhelming majority of whom in Australia are active participants. However, the great difference between this and most, perhaps all, other studies of participation is that this study is based on a state-wide census of sport participants, rather than a sample.

The results of this study are relevant to the fields of sport science and sport management in terms of providing evidence of participation trends over time and the differences across age groups. These findings can provide an evidence based to inform participation strategies and policies that aim at retaining participants.

We acknowledge some limitations to this study. Analyzing such a large but selective data set raises an issue of statistical inference methodology and interpretation. The data analyzed can either be regarded as representing a whole population (i.e., registered female participants in a particular sport in Victoria in the base year) or as a sample of some larger population (including other states and countries, other sports and/or other years). While the usual convention of citing statistical significance levels has been adopted in reporting the results of the statistical analyses, it is acknowledged that from the former perspective there is no inference beyond the observed data and so statistical significance really does not apply, and from the latter perspective, while the sample may or may not be representative, it is certainly not a random sample, and so the validity of the reported *p*-values is uncertain. In practical terms, this means that caution should be exercised regarding the generalizability of the results, particularly to other sports.

The strength of sport registration data is that it provides a more comprehensive and accurate record on which to base longitudinal analysis than does self-reported retrospective survey data. However, the trade-off is that such longitudinal analysis can generally only be conducted “within sports,” not “between sports” because privacy/anonymity requirements prevent cross-linking of the data for an individual across multiple sports. We acknowledge that gender and other contextual influences might make it difficult to generalize from this particular sport context to sport generally, but that would be true whatever sport context was studied. Furthermore, if a phenomenon is contextually influenced and hence heterogeneous, generalization is in principle difficult; mixing data from multiple settings might lead to aggregated results which do not apply very well to any of the particular settings. In such a situation, specificity might be regarded as a strength. One sport is a limitation, however 7 years of longitudinal data for analysis is a strength. We have demonstrated a method that could be applied to any sport for which sufficient historical data were available.

Another limitation relates to the definition of “commencement.” In a context such as survival analysis for a disease, initial diagnosis provides a well-defined starting point for the longitudinal tracking of each individual, but there is no such self-evident individual commencement point for the longitudinal analysis of an activity where participation can be ongoing or intermittent over long periods of the lifespan, and where historical data are unavailable. Instead, we viewed each individual's participation through a common temporal “window.” Even if retrospective reporting of participation were reliable (a dubious proposition), this study was based on an analysis of existing data, not on a survey in which we could ask about past participation. Without a filtering criterion, we would have had no way of knowing how long each individual had been playing. We had records for eight consecutive years, and we chose to use the data from the first year (2009) to select those who did not play in the first year but did play in the second year, which became our base year. Hence, we defined a cohort, of all ages, with a common “commencing” point in 2010 (albeit not necessarily first-time participation), and tracked their participation for 7 years. The trade-off for establishing this common starting point is a potential selection bias, because some of some of the longest-term participants, particularly those who had participated continually “year-in-year-out,” were excluded from the study (although any long-term intermittent participants who did not participate in 2009 were included). This bias is also age-dependent, since it is reasonable to expect that a high proportion of the younger participants were beginners, whereas most of the older participants would have been returning after a gap. A further limitation is that the sport has agreed to the publication of the results but not to identification of the sport.

## Conclusion

Overall 15% of the participants in the particular sport that was the subject of this study played continuously for 7 years. Retention, as defined by three different criteria, was consistently highest for those within the 4–9 year age group. There was a high percentage of people who played this sport, dropped-out and then returned. Further breakdown of ages demonstrated that an optimal starting age for participation in this sport, from the perspective of longevity or retention in the sport, may be 6–9 years, which is consistent with other recent recommendations (Eime et al., 2018).

Governing bodies such as Sport Australia, that have brought reducing physical inactivity levels in the population within their remit, could play an instrumental role in facilitating fundamental movement skill programs across all sport governing bodies, targeting young children in an effort to better prepare young children for entry into modified sport formats. Consideration needs to be given to the age appropriateness of sports programs for very young participants.

It is therefore recommended that sport policy and strategic practices have a specific focus on retention and not just on an increase in numbers, and that there is a redirection of funding for sport toward grass-roots participation. It is also recommended that sports organizations should (a) strategically focus on recruitment of participants into sport when they are developmentally ready (ages 6–9 years), and recruit younger participants into fundamental movement skills programs rather than organized sport s; (b) prioritize retention strategies and especially for those 15–19 years, which may involve the introduction of non-competitive sport products; and (c) develop strategies to provide supportive environments and sport offerings for those who want to enter the sport later in life and who do not have the sport-specific competencies relative to other peers who currently play.

## Data Availability Statement

The data is not able to be shared due to confidentiality agreements with the primary data holder. Data is not sharable at all, even to researchers. Please direct any enquiries regarding data to Rochelle Eime, r.eime@federation.edu.au.

## Ethics Statement

The studies involving human participants were reviewed and approved by Federation University Human Research Ethics Committee. Written informed consent from the participants' legal guardian/next of kin was not required to participate in this study in accordance with the national legislation and the institutional requirements.

## Author Contributions

RE contributed to the study design, interpretation of results, and manuscript conceptualization and preparation. MC and JH contributed to the study design, data management, statistical analysis and interpretation, and manuscript conceptualization and preparation. HW contributed to the interpretation of results and manuscript conceptualization and preparation. All authors have read and approved the final manuscript.

### Conflict of Interest

The authors declare that the research was conducted in the absence of any commercial or financial relationships that could be construed as a potential conflict of interest.
